# Effect of traditional Chinese Yijinjing exercise on hand dysfunction in rheumatoid arthritis patients: a randomized controlled trial

**DOI:** 10.3389/fmed.2024.1454982

**Published:** 2024-11-11

**Authors:** Tian Chang, Xieli Ma, Xun Gong, Congmin Xia, Quan Jiang, Rouman Zhang

**Affiliations:** ^1^Graduate College, Beijing University of Chinese Medicine, Beijing, China; ^2^Department of Medical Affairs, Guang’anmen Hospital, China Academy of Chinese Medical Sciences, Beijing, China

**Keywords:** rheumatoid arthritis, hand function, Yijinjing exercise, randomized controlled trial, Michigan Hand Outcomes Questionnaire

## Abstract

**Introduction:**

Rheumatoid arthritis (RA) patients often experience significant hand dysfunction. Yijinjing, a traditional Chinese exercise, has been recognized for its benefits to mind–body health. This study aimed to evaluate the efficacy and safety of Yijinjing in improving hand dysfunction among individuals with RA.

**Methods:**

This research was conducted as a single-center, outcome-blinded, randomized controlled trial. A total of 66 eligible RA participants were randomly assigned in a 1:1 ratio to either the Yijinjing exercise group (YJJG) with sessions conducted three times per week over a 12-week period or the control group (CG), which maintained ordinary activities. Various outcomes were assessed, including the Michigan Hand Outcomes Questionnaire (MHQ), handgrip strength, active range of motion (AROM), RA disease activity, the Health Assessment Questionnaire Disability Index (HAQ-DI), levels of anxiety and depression, and wrist ultrasound, all of which were collected at baseline and the week 12.

**Results:**

At the end of the 12 weeks, the YJJG demonstrated significant improvements in MHQ scores compared to the CG (*p* < 0.05), alongside enhancements in handgrip strength and AROM, specifically in wrist extension, radial deviation, and metacarpophalangeal flexion (*p* < 0.05). Wrist ultrasound scores did not exhibit a significant increase in the YJJG, meaning no inflammation aggravation (*p* > 0.05). Additionally, morning stiffness duration, Disease Activity Score 28-ESR (DAS28-ESR), erythrocyte sedimentation rate (ESR), and C-reactive protein (CRP) levels showed significant improvement in the YJJG (*p* < 0.05). Both groups reported enhancements in quality of life, as well as reductions in anxiety and depression scores, with the YJJG displaying greater improvements overall (*p* < 0.05). Importantly, no adverse events or significant abnormalities in vital signs were observed in either group.

**Conclusion:**

Yijinjing exercise may effectively enhance hand function, handgrip strength, and flexibility in RA patients with low disease activity. Furthermore, it appears to improve quality of life and reduce anxiety and depression without exacerbating joint inflammation.

**Clinical trial registration:**

https://clinicaltrials.gov/study/NCT05527158, Identifier NCT05527158.

## Graphical abstract

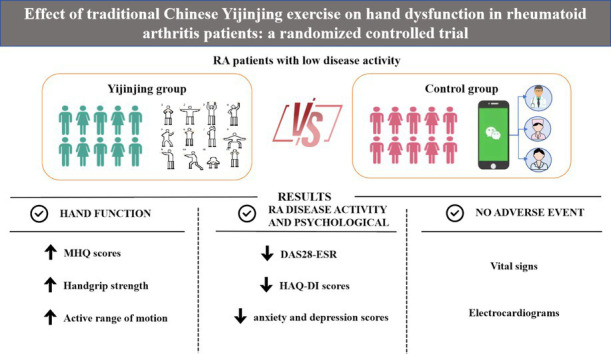

## Introduction

Rheumatoid arthritis (RA) is a chronic inflammatory disease characterized by severe disability, joint swelling, pain, and stiffness, ultimately leading to joint deformation, distortion, and a diminished quality of life (1). Approximately 0.2–1% of the global population suffers from RA, with a higher prevalence among females (2). The etiology and pathogenesis of RA remain incompletely understood. Pharmacological interventions are typically the first line of treatment. Despite advancements in biologics and disease-modifying antirheumatic drugs (DMARDs), which have significantly improved RA management in recent years, joint dysfunction-particularly in the hands-remains a major clinical challenge (3). Evidence suggests that approximately 70% of RA patients may experience bone erosion within 2 years of diagnosis (4). Furthermore, over 90% of RA cases initially manifest in the wrist and hand (5). Thus, addressing hand dysfunction in RA patients is paramount (6).

Although treat-to-target, patients fail to maintain sustained low disease activity or remission and hand dysfunction continues to plague RA patients. As a result, complementary and alternative medicine treatments for RA, such as acupuncture, physical exercise, and arthritic gloves, are gradually developed and emphasized. In the past few years, strengthening exercises for inflamed joints or flexion exercises against resistance have been questioned due to joint injury or excessive deformity concerns (7). Currently, active exercise is commonly recognized as a safe and effective treatment for RA. Professional rehabilitation guidance can alleviate dysfunction and improve quality of life in RA patients (8, 9). Consistent engagement in exercise received a strong recommendation in the 2022 American College of Rheumatology (ACR) guideline (10). Several meta-analyses have confirmed that exercise can relieve pain, improve somatic function, and enhance quality of life in RA patients, but it is still unclear what kind, how much, and how often they should take (11, 12).

Comparatively few clinical research aimed at hand function training for RA. The Strengthening and Stretching for Rheumatoid Arthritis of the Hand Trial (SARAH), an individually designed, progressive exercise program for the hand and arm, was conducted in a multicenter (*n* = 17), randomized, double-blind controlled trial (13). This exercise may reduce hand discomfort and strengthen hand function even if RA patients with acute small muscle atrophy and hand abnormalities (4, 14). It is also beneficial for daily life, work, and mood, potentially persisting for 12 months (15, 16). However, it is still unclear whether exercise can generate medium-and long-term benefits. It is necessary to carry out further high-quality research to investigate the benefit of exercise on hand function in RA (17).

Yijinjing is a multi-component and psychosomatic exercise based on traditional Chinese medicine theory. It is easy to learn, convenient, high-security, moderate-intensity, and combines static and dynamic movements with non-invasive external physiotherapy (18). Yi symbolizes change, Jin represents muscles, sinews, bones, and joints, and Jing means methods that integrate the body with mood (19). As a result, it offers a profitable complementary therapy for ameliorating physical function, regulating emotions, and relieving mental stress (20). Yijinjing has primarily focused on motor and cognitive function in stroke sequelae, as well as on osteoarthritis, ankylosing spondylitis, chronic schizophrenia (21, 22). Nevertheless, there are few studies on RA (23). Therefore, we conducted a randomized controlled trial to fill these gaps, with the main goal of improving hand function. We hypothesized that Yijinjing exercise would exert a positive impact on hand function in RA patients.

## Methods

### Participants

Participants were recruited from the rheumatology department at Guang’anmen Hospital, China Academy of Chinese Medical Sciences, from February 2022 to December 2022. The subjects met the following criteria: (1) met the 2010 ACR/European League Against Rheumatism (EULAR) classification criteria or the 1987 revised ACR classification criteria; (2) were aged 18–65, regardless of sex; (3) with low disease activity (DAS28-ESR ≤ 32); (4) with joint function in grades I to II and X-ray in stages I to II; (5) with a stable dose of DMARDs, biological agents, or glucocorticoids within 4 weeks prior to screening; (6) all patients were evaluated for hand dysfunction by a professional rheumatologist; and (7) volunteered to participate and cooperated to complete this study. The exclusion criteria were as follows: (1) had Yijinjing exercise experience within the last 3 months; (2) had severe joint deformity (eg. subluxation and severe ulnar deviation) or joint ankylosis; (3) had other diseases affecting limb function, such as trauma, fractures, infections, tumors, or congenital malformations; (4) had complications such as severe cardiovascular, brain, liver, lung, kidney, or hematopoietic system diseases; and (5) had moderate or severe cognitive impairment. All patients signed informed consent prior to baseline assessments for eligibility.

### Study design

This was a 12-week single-center randomized controlled trial in parallel. Sixty-six eligible patients were randomly assigned to receive the Yijinjing exercise (the YJJG group) or usual care control (the CG group) in a 1:1 ratio. The randomization was generated through a random number table by the staff not involved in recruitment and subsequently using sealed envelopes to assign. The outcome assessors were blinded to the allocation. All outcomes were measured at baseline and 12 weeks.

The study protocol, informed consent, and case report forms (CRFs) all met the Helsinki Declaration, and the enrollment, intervention, and measurement schedule adhered to SPIRIT’s requirements. This trial protocol has been registered in ClinicalTrials (NCT05527158) and approved by the Guang’anmen Hospital Research Ethics Committee, Chinese Academy of Chinese Medical Sciences [2022-002-KY-01].

### Sample size

The sample size was calculated by change in the Michigan Hand Outcomes Questionnaire score. Due to the minimal clinically important difference in the MHQ score is 11.0 (24), we assumed a between-group difference of 11.0 points, and a standard deviation of 12.5, with an alpha value of 0.05, and a test power of 90%. Considering a 20% shedding rate, we eventually enrolled at least 66 participants, 33 individuals in each group. The formula is as follows.


$$N_{1}=N_{2}=2{\left(\sigma \frac{t_{\alpha /2}+t_{\beta }}{\mu_{1}-\mu_{2}}\right)}^{2}$$

### Interventions

**Figure 1:**
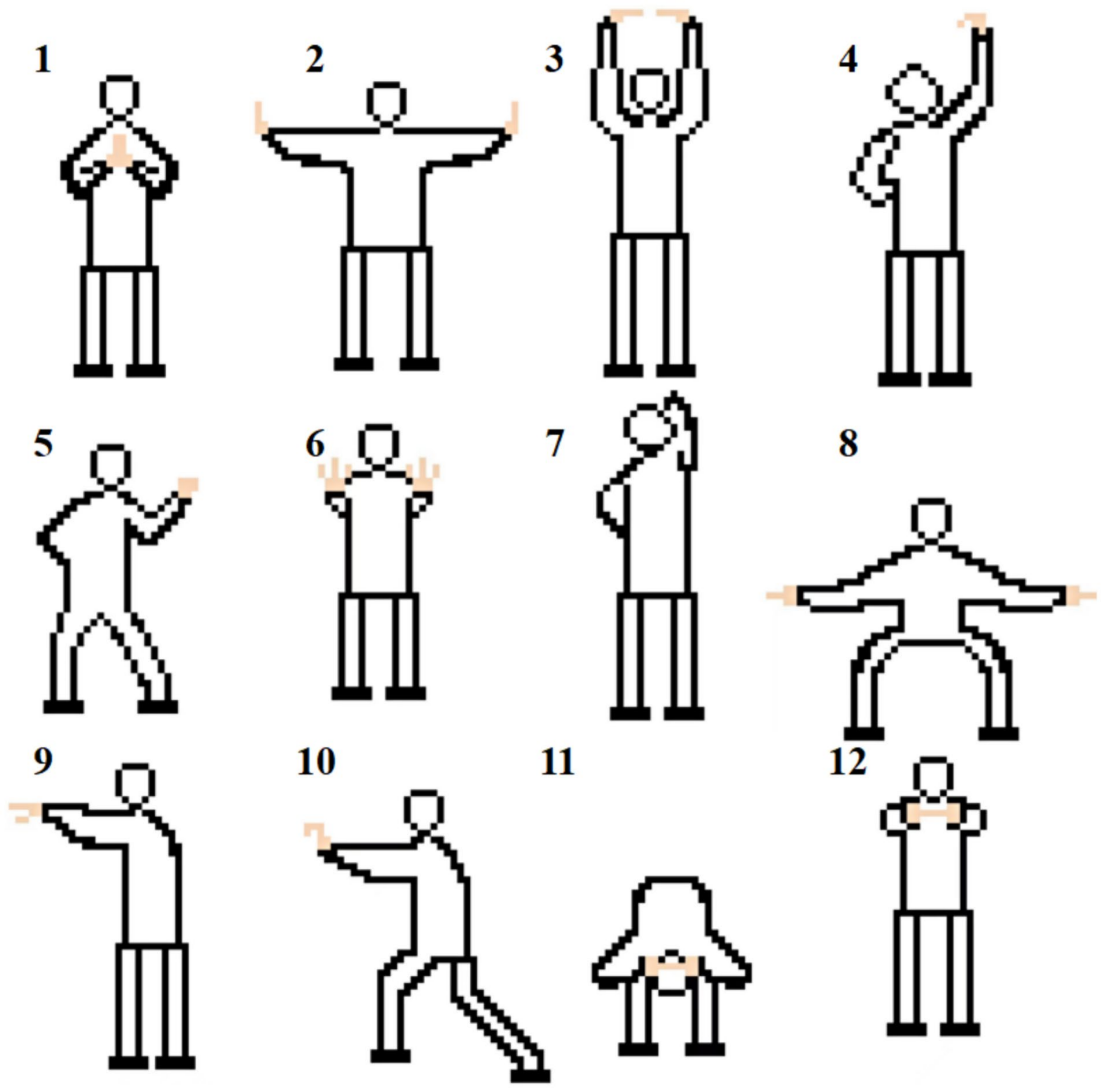
Twelve postures of Yijinjing exercise.

We advised patients to maintain regular daily activities but not to initiate any other new exercise. The necessity of physical activity and at-home exercise, daily preventive measures and awareness of RA were emphasized for all participants. All patients sustained stable pharmacological therapy for RA. The CG group received routine joint rehabilitation guidance, such as energy conservation and protection, without additional exercise interventions. We established *Wechat group* to engage health education and monitor their compliance. The YJJG group underwent Yijinjing exercise three times a week for 12 weeks (Tuesday, Thursday, Saturday, 8:00 p.m. to 9:00 p.m.). Each practice lasted 1 h, including three integral Yijinjing exercises with a five-minute break after each exercise. The Yijinjing, compiled by the State Sports General Administration of China and consisted of 12 operational postures, was recommended as the standard intervention (Figure 1; Table 1). Professional coach with at least 5 years of experience carried out action decomposition teaching before the formal practice started. During the exercise, we paid attention to the patient’s hand function, especially flexion and extension of the wrist and interphalangeal joints. In addition, we concentrated on the center of gravity movement and assisted breathing naturally. The exercise was undertaken in the form of online *Tencent meetings*. The researchers evaluated the attendance of meetings and instructed patients to keep self-exercise report records. As long as the attendance record was >65% during the 3 months, the experiment was deemed complete (Supplementary Table S1). At later 12 weeks, the patients were not asked to repeat the practice at home in both groups. They can keep practice based on their own preference.

**Table 1:** Exercise intervention program.

Warm up	Yijinjing exercise	Cool down
Activity: Wrist rotation flexion and extension; chest expansion; waist rotation; ankle rotation flexion and extension Duration: 5 min Intensity: mild	Activity: Twelve postures of Yijinjing exercise Frequency: 3 times a week Intensity: moderate Duration/time: 50 min Repetitions: 3 times Type: aerobic exercise, flexibility exercise Progression: Gradually increase to 3 times according to the individual situation, 2 times are allowed	Activity: Wrist rotation flexion and extension; chest expansion; waist rotation; ankle rotation flexion and extension Duration: 5 min Intensity: mild

#### Outcome measurements

##### Primary outcome

The Michigan Hand Outcomes Questionnaire (MHQ) consists of 37 questions divided into the following six categories: (1) overall hand function; (2) activities of daily living; (3) work performance; (4) pain; (5) esthetics; and (6) patient satisfaction with hand function, measured individually for the left and right hands (25). The MHQ scale was chosen as the primary outcome due to its comprehensive assessment of hand function. It is an extensively used hand-specific outcome tool in clinical chronic hand illnesses with strong reliability and validity (26). It is designed to capture the patient’s subjective experience of their hand condition, which is crucial in evaluating the impact of treatments on daily life and quality of life. Furthermore, the MHQ allows for separate evaluation of the left and right hand, which is particularly beneficial in conditions where hand dominance and asymmetry in disease severity.

#### Secondary outcomes

##### Handgrip strength and flexibility

Handgrip strength can reflect hand strength. The use of electronic grippers is more sensitive and easier for patients with weaker grip strength (27). Hence, an electronic gripper (CAMRY, TXUT-013, Guang dong, China) was used to assess handgrip strength (kg). The subjects grabbed with the highest possible force while not suffering any pain or discomfort when seated, with their shoulders not twisted, their elbows bent at a 90-degree angle, and their forearms in a neutral position (28). It was carried out three times with an interval of 15 s, and set the average as the final result. One measurement was acceptable for those with visibly painful joints (6).

The active range of motion (AROM) reflects hand flexibility. Reduced hand ROM is closely related to patient functional disability (29). We used a protractor to judge the AROM. First, we determined the joint and fixed axis and then rotated the moving axis relative to the zero-degree position to calculate the wrist flexion, extension, ulnar deviation, radial deviation, MCP flexion, and PIP flexion angles. The subjects must use their muscular power to move.

#### RA disease activity

The duration (in minutes) of morning stiffness was gauged by the patients’ self-reports. The visual analog scale (VAS) indicated the pain in RA, with 0 signifying “no pain” and 10 signifying “the worst intolerable pain” (30). We evaluated the number of swollen, painful, and deformed joints among the 28 peripheral joints. Rheumatoid factor (RF), the erythrocyte sedimentation rate (ESR), and C-reactive protein (CRP) were detected and recorded. Thus, we assessed RA disease activity using the internationally recognized DAS28-ESR.

#### Quality of life

The Health Assessment Questionnaire Disability Index (HAQ-DI) has been thoroughly validated and measured in RA (31). The scale contains 20 questions about daily activities such as dressing, grooming, arising, eating, walking, maintaining hygiene, reaching and gripping (32).

#### Psychological condition

The self-rating depression scale (SDS) was used to measure the severity of depression; higher scores reflect symptom severity (33). The self-rating anxiety scale (SAS) mainly consists of 20 items to assess the intensity of anxiety.

#### Wrist ultrasound

Two professionally trained musculoskeletal ultrasound physicians evaluated the conditions of synovitis, tenosynovitis, and bone erosion on the wrist joint. According to the European League against Rheumatism-Rheumatology Outcome Measurement (EULAR-OMERACT) score (34), synovitis, tenosynovitis, and bone erosion of the same wrist joint were graded at baseline and 12 weeks.

#### Safety assessment

Electrocardiograms (ECGs) were performed, along with the individuals’ vital signs (blood pressure, respiration, heart rate, and pulse), and adverse events were recorded.

### Statistical analysis

SPSS 26.0 statistical software (IBM. US) was used for data analysis, and *p* < 0.05 was considered to indicate statistical significance. Continuous variables are described as the means ± standard deviations for normally distributed data or medians (25, 75%) for non-normally distributed data, while categorical data are expressed as frequencies and percentages. For differences between groups, an independent sample *T*-test (normal, homogeneity of variance) or Wilcoxon Mann–Whitney test (non-normal, heterogeneity of variance) was used for continuous data, while the Chi-square test or Fisher’ s exact test was used for categorical data and grade data. Time differences (baseline, 12 weeks) were defined as intra-subject, and the presence or absence of Yijinjing exercise was defined as between-subject factor. In terms of intra-group comparisons, the paired-sample t-test or Wilcoxon Mann–Whitney test (non-normal) was used for the continuous data, while the McNemar test or the McNemar-Bowker test was used for the categorical data.

## Results

### Participants and baseline characteristics

**Figure 2:**
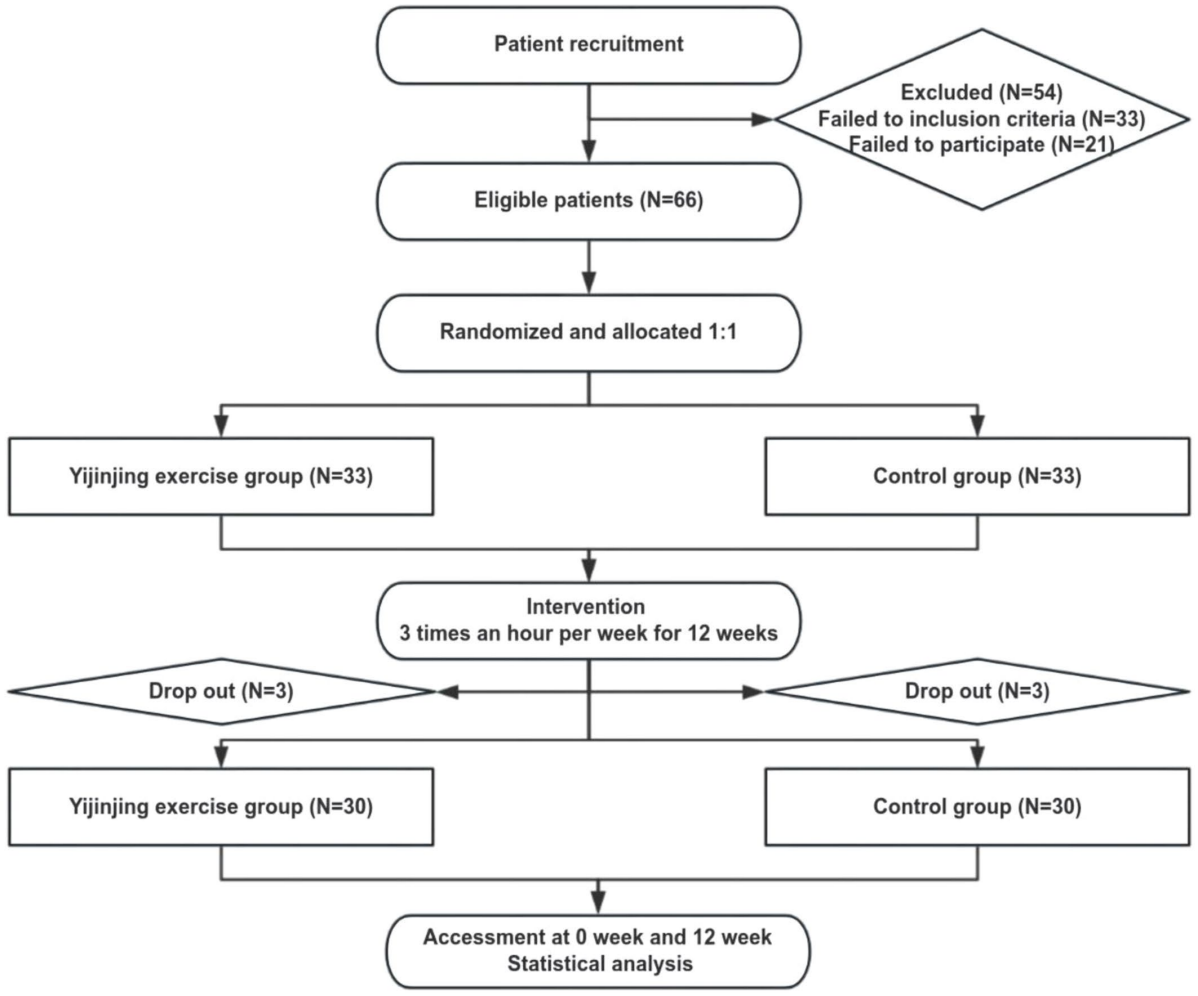
Participant flow diagram.

One hundred twenty patients were screened for eligibility and 66 patients were eventually randomized. Six subjects dropped out due to loss to follow up, and 60 subjects finally completed the trial (YJJG: *n* = 30, CG: *n* = 30). The entire trial flow diagram is shown in Figure 2. 95% of subjects were female, and the mean ages were 54.80 ± 8.50 and 51.63 ± 10.63 years, respectively, which is consistent with the prevalence of RA in middle-aged and elderly females. At baseline, they were comparable between the two groups in terms of age, sex, height, weight, MHQ score, handgrip strength, AROM, RA disease activity (DAS28-ESR), ECG, and so on (*p* > 0.05). The medical treatment history for RA over the past 3 months was similar between the two groups. The DAS28-ESR of both groups was <3.2, and the disease activity was relatively stable (Table 2).

**Table 2:** Baseline characteristics of participants.

Characteristic	Control Group (*n* = 30)	Yijinjing Group (*n* = 30)	*p* value
Age (year)	51.63 ± 10.63	54.80 ± 8.50	0.207
Gender (female/male), n/n	28/2	29/1	1.000
Height (cm)	161.57 ± 5.85	161.82 ± 4.80	0.875
Weight (kg)	59.37 ± 8.81	57.57 ± 7.27	0.392
MHQ score (range 0–100)	49.58 ± 10.48	51.48 ± 8.36	0.442
Handgrip strength (kg, right)	17.89 ± 6.70	19.07 ± 5.96	0.474
Handgrip strength (kg, left)	16.91 ± 6.38	18.91 ± 6.79	0.246
Wrist/finger AROM (°)			
Wrist extension (right)	53.5 (41.75, 56.25)	53.0 (46.25, 58.0)	0.773
Wrist extension (left)	54.0 (40.0, 60.0)	54.0 (51.5, 60.0)	0.149
Wrist flexion (right)	57.0 (36.5, 66.0)	57.0 (42.0, 64.0)	0.982
Wrist flexion (left)	56.0 (40.5, 64.0)	56.0 (46.0, 62.5)	0.750
Wrist ulnar deviation (right)	30.0 (25.5, 35.25)	30.0 (23.0, 32.0)	0.435
Wrist ulnar deviation (left)	30.0 (26.0, 36.0)	30.0 (23.5, 34.0)	0.457
Wrist radial deviation (right)	13.63 ± 5.20	14.20 ± 3.94	0.636
Wrist radial deviation (right)	12.5 (10.0, 18.0)	14.0 (12.0, 16.5)	0.317
MCP flexion (right)	80.0 (73.0, 86.0)	80.0 (71.5, 84.0)	0.503
MCP flexion (left)	80.0 (78.0, 84.5)	81.0 (78.0, 84.0)	0.771
PIP flexion (right)	89.93 ± 10.78	85.67 ± 10.09	0.119
PIP flexion (left)	92.0 (87.5, 96.5)	90.0 (80.0, 92.5)	0.150
finger extension (cm, right)	3.0 (1.73, 4.0)	3.15 (3.0, 4.0)	0.294
finger extension (cm, left)	3.65 (2.08, 4.43)	3.8 (3.5, 4.28)	0.369
Morning stiffness duration (min)	15 (10, 20)	15 (10, 30)	0.214
DAS28-ESR (score)	2.97 ± 0.85	2.85 ± 0.82	0.674
ECG abnormal (*n*, %)	8 (26.67%)	11 (36.67%)	0.405
Previous treatments within 3 months (*n*, %)			0.876
Chinese herbal decoction	27 (90.00%)	28 (93.33%)	
Methotrexate	5 (16.67%)	10 (33.33%)	
Leflunomide	5 (16.67%)	4 (13.33%)	
Iguratimod	1 (3.33%)	3 (10.00%)	
Hydroxychloroquine	1 (3.33%)	1 (3.33%)	
Tofacitinib	7 (23.33%)	4 (13.33%)	
bDMARDs	5 (16.67%)	4 (13.33%)	
NSAIDs	1 (3.33%)	1 (3.33%)	
Glucocorticoids	0 (0)	1 (3.33%)	
Tripterygium Glycosides	10 (33.33%)	12 (40.00%)	
Chinese patent medicine	2 (6.67%)	3 (10.00%)	

The values are expressed as the means ± SD or medians (25, 75%) (if data are not normally distributed). MHQ, Michigan Hand Outcomes Questionnaire; AROM, active range of motion; MCP, metacarpophalangeal joint; PIP, proximal interphalangeal joint; DAS28-ESR, disease activity score in 28 joints-erythrocyte sedimentation rate; ECG, electrocardiogram, abnormal but not clinically significant; bDMARDs, biological disease modifying anti-rheumatic drugs; NSAIDs, nonsteroidal anti-inflammatory drugs; *p* > 0.05 indicated no significant difference between groups at baseline. MCP/PIP flexion was calculated by means of MCP2-4 and PIP2-4.

### Effect of Yijinjing on hand function

Hand function including the MHQ score, handgrip strength, and AROM, was definitely ameliorated in the YJJG group compared with the CG group at 12 weeks. Not only in MHQ total score, but also six MHQ sub-scales, the YJJG group performed better than the CG group, which is clinically significant (*p* < 0.05). In terms of handgrip strength, there was an increase more than 1.0 kg for both the left and right hands in YJJG group (*p* < 0.05), while the CG group did not significantly improve compared with baseline (*p* > 0.05). Additionally, in the YJJG, all measures of AROM significantly increased by 6–10° (*p* < 0.05), whereas in the CG, the increase ranged from 1 to 4° compared with baseline. Wrist extension, wrist radial deviation, and MCP flexion in the YJJG group were greater than those in the CG (*p* < 0.05), but no significant changes in PIP flexion, wrist ulnar deviation, and left wrist flexion were observed between the two groups (*p* > 0.05). The wrist ultrasound score showed a slight decrease in the YJJG and a mild increase in the CG at 12 weeks with no statistical significance (*p* > 0.05). Additionally, the Yijinjing exercise did not exacerbate bone erosion and inflammation (Table 3).

**Table 3:** Effect of Yijinjing on hand function.

Hand function	Baseline		Week 12		*p* value (within-group)		*p* value (between-group)
	CG (*n* = 30)	YJJG (*n* = 30)	CG (*n* = 30)	YJJG (*n* = 30)	CG	YJJG	
MHQ score	49.58 ± 10.48	51.48 ± 8.36	55.04 ± 7.57	65.57 ± 5.86	<0.001	<0.001	**<0.001**
Handgrip strength-R (kg)	17.89 ± 6.70	19.07 ± 5.96	18.08 ± 6.37	20.26 ± 6.20	0.323	0.002	**<0.001**
Handgrip strength-L (kg)	16.91 ± 6.38	18.91 ± 6.79	17.16 ± 5.77	20.62 ± 5.93	0.366	<0.001	**<0.001**
Total ultrasound score	3 (2, 5)	3 (1, 5)	4 (2, 5)	2 (1, 4.5)	0.092	0.057	**0.027**
Synovitis grade	1 (1, 2)	1 (1, 2)	2 (1, 3)	1 (0, 2)	0.334	0.145	0.052
Peritendinitis grade	0 (0, 0)	0 (0, 1)	0 (0, 1)	0 (0, 1)	0.751	0.238	0.075
Bone erosion grade	2 (0, 3)	0 (0, 3)	2 (0, 3)	0 (0, 3)	0.102	0.890	0.386
Wrist extension-R	53.5 (41.75, 56.25)	53.0 (46.25, 58.0)	54.5 (39.5, 58.5)	62.0 (54.75, 66.25)	0.002	<0.001	**0.002**
Wrist extension-L	49.63 ± 12.31	54.0 (51.5, 60.0)	52.20 ± 12.21	65.0 (60.0, 70.0)	<0.001	<0.001	**<0.001**
Wrist flexion-R	57.0 (36.5, 66.0)	57.0 (42.0, 64.0)	59.0 (39.75, 64.0)	66.0 (56.0, 70.0)	0.009	<0.001	**0.030**
Wrist flexion-L	56.0 (40.5, 64.0)	54.0 (51.5, 60.0)	58.0 (41.5, 64.5)	62.5 (52.0, 70.0)	0.002	<0.001	0.071
Wrist radial deviation-R	14.0 (8.0, 18.0)	14.0 (11.5, 18.0)	16.0 (10.0, 18.5)	20.0 (18.0, 20.0)	<0.001	<0.001	**0.002**
Wrist radial deviation-L	12.5 (10.0, 18.0)	14.0 (12.0, 16.5)	14.0 (11.5, 18.5)	18.0 (16.0, 20.0)	0.003	<0.001	**0.002**
Wrist ulnar deviation-R	30.0 (25.5, 35.25)	30.0 (23.0, 32.0)	30.0 (25.5, 36.0)	34.0 (29.5, 38.0)	0.121	<0.001	0.105
Wrist ulnar deviation-L	30.0 (26.0, 36.0)	28.87 ± 5.84	31.0 (26.0, 36.0)	32.60 ± 4.21	0.181	<0.001	0.348
MCP flexion-R	79.00 ± 7.56	80.0 (71.5, 84.0)	81.93 ± 5.34	88.0 (85.5, 90.0)	0.002	<0.001	**<0.001**
MCP flexion-L	80.40 ± 6.27	81.0 (78.0, 84.0)	82.53 ± 4.36	88.0 (84.0, 90.0)	0.002	<0.001	**0.002**
PIP flexion-R	92.0 (85.5, 98.0)	90.0 (78.0, 94.0)	94.0 (88.0, 98.0)	96.0 (93.5, 98.5)	0.006	<0.001	0.109
PIP flexion-L	92.0 (87.5, 96.5)	90.0 (80.0, 92.5)	94.0 (87.5, 98.5)	96.0 (92.0, 98.5)	<0.001	<0.001	0.104
finger extension-R (cm)	2.85 ± 1.32	3.15 (3.0, 4.0)	3.08 ± 1.29	3.9 (3.0, 4.58)	0.023	<0.001	**0.018**
finger extension-L (cm)	3.65 (2.075, 4.425)	3.8 (3.5, 4.28)	3.75 (2.25, 4.80)	4.5 (4.0, 4.65)	0.010	<0.001	0.056

The values are expressed as the means ± SDs or medians (25, 75%) (if data are not normally distributed). CG, control group; YJJG, Yijinjing group; MHQ, Michigan Hand Outcomes Questionnaire; R, right; L, left; AROM, active range of motion; MCP, metacarpophalangeal joint; PIP, proximal interphalangeal joint. *p* < 0.05 indicated a significant difference. Bold values in table indicate statistically significant differences between groups.

### Effect of Yijinjing on RA disease activity

After 12 weeks of Yijinjing exercise, the VAS score, morning stiffness duration, number of tender joints, number of swollen joints, and DAS28-ESR in the YJJG group were significantly different from baseline (*p* < 0.05). However, there was no significant decrease in RF, ESR, and CRP (*p* > 0.05). No evidence of within-group differences in the CG was found, except for the VAS score and morning stiffness minutes (*p* < 0.05). Compared with the CG, the VAS score, morning stiffness minutes, DAS28-ESR, ESR, and CRP in the YJJG group significantly improved (*p* < 0.05). More importantly, the disease activity in the YJJG turned into remission (Table 4).

**Table 4:** Effect of Yijinjing on RA disease activity.

RA disease activity	Baseline		Week 12		*p* value (within-group)		*p* value (between-group)
	CG (*n* = 30)	YJJG (*n* = 30)	CG (*n* = 30)	YJJG (*n* = 30)	CG	YJJG	
PGA	30 (20, 36.25)	30 (20, 30)	20 (10, 30)	10 (10, 11.25)	<0.001	<0.001	**<0.001**
MSD (min)	15 (10, 20)	15 (10, 30)	10 (8.8, 11.3)	5 (0, 5)	0.003	<0.001	**<0.001**
TJC	1 (0, 2)	1 (0.75, 3.0)	0.5 (0, 2)	0 (0, 2)	0.069	0.001	0.432
SJC	0 (0, 0)	0 (0, 0)	0 (0, 0)	0 (0, 0)	0.429	0.025	0.078
RF (umol/L)	97.69 (32.54, 207.67)	53.7 (27.31, 143.48)	99.52 (37.24, 233.97)	62.38 (37.2, 132.56)	0.884	0.673	0.099
ESR (mm/h)	16.0 (11.5, 25.0)	13.5 (7.0, 23.0)	23 (14, 33)	10 (7, 18.75)	0.179	0.346	**0.007**
CRP (mg/L)	0.64 (0.5, 2.01)	0.5 (0.5, 1.44)	2.57 (0.5, 4.59)	0.5 (0.5, 0.5)	0.030	0.463	**<0.001**
DAS28-ESR	2.97 ± 0.85	2.85 ± 0.82	2.83 ± 0.67	2.15 ± 0.83	0.621	0.001	**0.002**

The values are expressed as the means ± SDs or medians (25, 75%) (if data are not normally distributed). CG, control group; YJJG, Yijinjing group; PGA, Patient’s Gobal assessment of disease activity on a VAS 0–100 mm; MSD, Morning stiffness duration; TJC, Tender joint count; SJC, Swollen joint count; RF, Rheumatoid factor; ESR, Erythrocyte sedimentation rate; CRP, C-reactive protein; DAS28-ESR, Disease activity score in 28 joints on erythrocyte sedimentation rate. *p* < 0.05 indicated a significant difference. Bold values in table indicate statistically significant differences between groups.

### Effect of Yijinjing on function and mental condition

Regarding dysfunction in daily activities, we observed a decline in HAQ-DI scores in both groups compared to baseline, with the YJJG showing superior improvement (*p* < 0.05). In terms of mental condition, the reductions in anxiety and depression scores from baseline to week 12 were significant (*p* < 0.05). Furthermore, no serious adverse events were reported in either the YJJG or the CG, indicating that the YJJG was as safe as the CG (Table 5).

**Table 5:** Effect of Yijinjing on function, mental health and safety.

	Baseline		Week 12		*p* value (within–group)		*p* value (between–group)
	CG (*n* = 30)	YJJG (*n* = 30)	CG (*n* = 30)	YJJG (*n* = 30)	CG	YJJG	
HAQ–DI score	0.13 (0, 0.75)	0.13 (0, 0.38)	0.13 (0, 0.5)	0 (0, 0)	0.003	<0.001	**0.036**
SAS score	37.5 (30.94, 45.31)	39.38 (32.5, 48.75)	33.75 (27.5, 38.75)	30.0 (27.5, 35.31)	<0.001	<0.001	**0.004**
SDS score	35.0 (28.44, 44.06)	35.0 (31.25, 46.56)	31.25 (27.5, 40.0)	28.13 (26.3, 35.3)	<0.001	<0.001	**0.007**
Systolic pressure (mmHg)	119.00 ± 5.82	119.20 ± 4.87	116 (116, 122)	116 (115, 122)	–	–	0.621
Diastolic pressure (mmHg)	74 (72, 76)	72 (69, 76)	74 (72, 78)	74 (72, 77)	–	–	0.885
Pulse (counts/min)	72.43 ± 4.17	73.76 ± 6.19	72.22 ± 3.77	72.00 ± 4.97	–	–	0.867
Temperature (°C)	36.2 (36.2, 36.3)	36.2 (36.1, 36.3)	36.3 (36.2, 36.3)	36.2 (36.1, 36.3)	–	–	0.252
Respiration (counts/min)	17 (16, 18)	16 (16, 17)	16 (16, 18)	16 (16, 18)	–	–	0.383
ECG abnormal (*n*, %)	8 (26.67%)	11 (36.67%)	9 (30.0%)	13 (43.3%)	–	–	0.284

The values are expressed as the means ± SDs or medians (25, 75%) (if data are not normally distributed). CG, control group; YJJG, Yijinjing group; HAQ-DI, Health Assessment Questionnaire Disability Index; SAS, Self-rating Anxiety Scale; SDS, Self-rating Depression Scale; ECG, Electrocardiogram. *p* < 0.05 indicated a significant difference. Bold values in table indicate statistically significant differences between groups.

## Discussion

In 2022, the ACR recommended hand exercises to improve mobility and strength, with low certainty evidence suggesting that mind–body exercise can improve physical function. As far as we know, this is the first randomized controlled trial aimed to evaluate the effect of Yijinjing on hand dysfunction in patients with rheumatoid arthritis (RA). It showed an improvement in hand function and enhanced handgrip strength and flexibility (active range of motion) in RA patients with low disease activity with a potential to ameliorate quality of life and alleviate anxiety and depression.

Yijinjing can improve hand function in RA patients. Due to successive progressive joint erosion, RA often leads to functional disability, typically resulting in hand dysfunction (35). It showed that MHQ scores moderately correlate with disease activity, in which higher DAS28 scores tended to correlate with lower MHQ scores, indicating worse hand function (36). Our study revealed that most patients with low disease activity or remission were not satisfied with their hand function even without moderate or severe joint pain. The MHQ total and each subscale score indicated that Yijinjing exercise for 12 weeks effectively ameliorated the hand function. This finding was consistent with previous studies (4) in strengthening and stretching for rheumatoid arthritis of the hand (SARAH) exercise. The Yijinjing exercise is highly similar to the SARAH program. For example, the first posture *Wei tuo xian chu* requires putting your hands together before your chest and maintaining as far as possible 90° wrist extension to exercise the wrist fully. Our study preliminarily showed that Yijinjing exercise effectively improves MHQ hand function in RA patients.

Yijinjing can enhance handgrip strength and flexibility in RA patients. Handgrip strength is critical for assessing hand function and predicting disability and joint impairment in RA (37, 38). The active range of motion (AROM) reflects hand flexibility quantitatively (39). High disease activity, more pain, severe dysfunction, hand disability, and bone erosion in RA patients have been associated with low grip strength (40) and worse AROM (41). For healthy people, the grip strength in a neutral position in the dominant hand was 29.1 kg, with a wrist flexion of 79.7°, a wrist extension of 74.4°, a pronation position ulnar deviation of 32.8° and a radial deviation of 21.1° in Stacy Fan’s study (42). However, for RAs with high disease activity, the grip strength was 11.4 kg (43), with wrist flexion of 38.7°, wrist extension of 35.2°, ulnar deviation of 29.7° and radial deviation of 13.1° (29). In terms of our study, we included 95% RA with low disease activity. The grip strength at baseline was less than 20 kg, which was weaker than healthy individuals but better than RA with high disease activity, showing a similar trend in AROM, which may be related to less pain. Our study revealed that handgrip strength and AROM were significantly greater in the YJJG than CG at 12 weeks, indicating Yijinjing exercise effectively improves hand muscle strength and ameliorates hand flexibility. This finding was consistent with Mark A Williams (4), but due to the different use of grip devices, we did not measure the pinch grip force, which was one of the limitations of our study. The reason about AROM did not reach healthy can be explained as follows. First, this study was carried out in RA patients with constant hand dysfunction. Second, even though the RA is relatively stable, there still exists subclinical synovitis under ultrasound or MRI. In addition, this may be related to insufficient length of intervention. Besides, there is no standard method to measure RA joint mobility. Naoto Ienaga (44) developed a smartphone-based system to assess the AROM of wrist. Whose accuracy was within a clinically usable error range. The Yijinjing exercise contains multiple hand or wrist movements to effectively promote muscular strength and dexterity in hand.

Yijinjing did not increase inflammation in RA patients. As recommended by the EULAR (45), the semi-quantitative grading of musculoskeletal ultrasound is more sensitive for diagnosing subclinical synovitis in wrist and hand (46). This study revealed that subclinical synovitis and tenosynovitis were present in both groups at baseline. Although no significant difference was found between two groups, a gradual improvement was noted in YJJG, indicating that Yijinjing does not exacerbate synovitis and is safe for RA. A prospective intervention study made clear that strengthening exercise is beneficial for enhancing the cross-sectional area of the rectus femoris via ultrasonography (47). In addition, training did not increase blood flow on ultrasound Doppler induced by inflammation, indicating that it had no adverse effect on RA (48). Therefore, it is necessary to introduce ultrasound as an evaluation to monitor the safety of exercise in RA. If the ultrasound score significantly increases, it hints at reducing intensity or stopping temporarily.

The morning stiffness duration, patient global assessment of disease activity, TJC, SJC, RF, CRP, and ESR are associated with RA disease activity. DAS28-ESR < 2.6, as a remission criterion, may be appropriate for most patients in the clinic (49). At 12 weeks the Yijinjing further reduced the disease activity of stable RA, gradually run up to remission, and did not aggravate joint inflammation. The effect of aerobic exercise on the prevention and treatment of RA dysfunction and disease activity has been gradually recognized. A study affirmed that aerobic and resistance exercise improved physical fitness in terms of aerobic capacity, endurance, and strength in older adults with RA (50). Tai Chi is safe for RA patients, but more evidence is needed to improve physical function and pain with active RA (51). Future researchers could attach importance to exercise in ameliorating function with moderate/severe disease activity RA. Exercise not only improved function but also controled inflammation, and the best duration and intensity of exercise should be explored in the future.

Yijinjing is beneficial for mind–body function in RA patients. RA with dysfunction often affects quality of life (52). Depression and anxiety are common comorbidities of RA, with the prevalence of depression varying from 14 to 48% (53), and the prevalence of anxiety was 62.1%, which is even greater than depression (54). Therefore, we should not only control the disease activity but also pay attention to improving quality of life and mental health. It showed that the HAQ-DI in the YJJG as well as anxiety and depression scores were obviously reduced in our study. During the follow-up, most patients said they were willing to exercise, but they were afraid the strenuous exercise would aggravate joint disorder, and they were irritable or depressed in the long term, which not only affected mental health but also further influenced body function. Yijinjing exercise made the patients sweat slightly, relaxed mind and body, and patients were willing to maintain. This study provided a rehabilitation treatment for RA patients with psychosomatic benefits.

Rheumatoid arthritis primarily presents as symmetric joint swelling, pain, and stiffness, most commonly affecting the hands and wrists, and can lead to joint deformity and loss of function. Yijinjing exercise contains a lot of fine hand movements, and acts on muscles, sinews, bones, and joints. It helps promote blood circulation, improve finger flexibility and coordination, and alleviate symptoms such as morning stiffness, localized inflammation, and pain. This contributes to maintain hand function, address motion difficulties caused by arthritis, and enhance the patient’s quality of life. Additionally, Yijinjing exercise can help reduce psychological stress and boost the patient’s confidence in managing the disease. Thus, Yijinjing offers a holistic approach, improving not just hand function but also overall well-being and mental health. It combines gentle movements with meditation and breathing techniques, which may lead to better compliance compared to isolated functional exercises. Moreover, Yijinjing appears to be safe for RA patients, without exacerbating inflammation.

Due to outbreak of COVID-19 epidemic, we choose instruct and monitor practice through *online Tencent meetings* via virtual mode over the offline face to face. It showed the adherence is acceptable. Online practice breaks the geographical limitations, saving patients expenses and time when seeking face to face care. Additionally, online practice supports simultaneous participation for multiple individuals, which can enhance patient engagement, improve compliance, and boost the effect.

Our study adds the growing literature about the cost-effective and non-toxic interventions to help improve the hand function in RA. However, it suffers from some limitations. First, the participant sample size is low that more data is required from more highly powered studies. Second, our study was only single-blinded because the characteristic of the intervention which means we could not control for non-specific effects. Third, there was no follow-up period, so we have no idea how quickly participants returned to baseline levels of all the study parameters. Besides, our study focused on RA patients with low disease activity. Subsequent research can further explore the effect in RA with medium and high disease activity. Last, our study were lack of an active control group. In the future, further multi-center RCTs should be carried out, and a active control, such as SARAH, could be set up to obtain more evidence.

In conclusion, Yijinjing exercise can improve hand function and enhance handgrip strength and flexibility in RA patients with low disease activity while ameliorating quality of life and alleviating anxiety and depression while safe and not aggravating joint inflammation.

## Data Availability

The raw data supporting the conclusions of this article will be made available by the authors, without undue reservation.
