# Supplementary material for: Effect of traditional Chinese Yijinjing exercise on hand dysfunction in rheumatoid arthritis patients: a randomized controlled trial
Source: Front Med (Lausanne). 2024 Nov 11;11:1454982. doi: 10.3389/fmed.2024.1454982 (PMC11586198; doi:10.3389/fmed.2024.1454982)
Supplement: Supplementary file 1 [file Table_1.docx]

**Supplement 1 The attendance of Yijinjing exercise**

| **Random Number** | **Name** | **Week 0- week 4** | | | | | | | | | | | | | |  | **Week 4-week 8** | | | | | | | | | | | |  | **Week 8-week 12** | | | | | | | | | | | | | **Attendance Rate** |
| --- | --- | --- | --- | --- | --- | --- | --- | --- | --- | --- | --- | --- | --- | --- | --- | --- | --- | --- | --- | --- | --- | --- | --- | --- | --- | --- | --- | --- | --- | --- | --- | --- | --- | --- | --- | --- | --- | --- | --- | --- | --- | --- | --- |
| 1 | GAYL | 2022.3.1 | 3.3 | 3.5 | 3.8 | 3.10 | 3.13 | 3.15 | 3.17 | 3.19 | 3.22 | 3.24 | 3.26 | 3.29 | 3.31 |  | 4.3 | 4.7 | 4.9 | 4.12 | 4.14 | 4.16 | 4.19 | 4.21 | 4.23 | 4.26 | 4.28 | 4.30 |  | 5.3 | 5.5 | × | × | × | × | × | × | × | × | × | × | × | 71.80% |
| 3 | REGH | 3..1 | 3.3 | 3.5 | × | × | × | × | × | × | × | × | × | × | × |  | × | × | × | × | × | × | × | × | × | × | × | × |  | × | × | × | × | × | × | × | × | × | × | × | × | × | 7.70% |
| 4 | DIRX | 3.1 | 3.3 | 3.5 | 3.8 | 3.10 | 3.13 | 3.15 | 3.17 | 3.19 | 3.22 | 3.24 | 3.26 | 3.29 | 3.31 |  | × | 4.7 | × | 4.12 | 4.14 | 4.16 | 4.19 | 4.21 | × | 4.26 | 4.28 | 4.30 |  | 5.3 | 5.5 | 5.8 | 5.10 | 5.12 | 5.14 | 5.17 | 5.19 | 5.21 | 5.24 | 5.26 | 5.28 | 5.31 | 92.30% |
| 5 | LISZ | 3.1 | × | × | 3.8 | 3.10 | 3.13 | 3.15 | 3.17 | 3.19 | 3.22 | 3.24 | 3.26 | × | 3.31 |  | 4.3 | 4.7 | 4.9 | 4.12 | 4.14 | 4.16 | 4.19 | 4.21 | 4.23 | 4.26 | 4.28 | 4.30 |  | 5.3 | 5.5 | 5.8 | 5.10 | × | 5.14 | 5.17 | 5.19 | 5.21 | × | 5.26 | 5.28 | 5.31 | 87.20% |
| 8 | GARZ | 4.19 | 4.21 | 4.23 | × | 4.28 | 4.30 | 5.3 | 5.5 | 5.8 | 5.10 | 5.12 | 5.14 | 5.17 | 5.19 |  | 5.21 | 5.24 | 5.26 | 5.28 | 5.31 | 6.2 | 6.5 | 6.7 | 6.9 | 6.11 | 6.14 | 6.16 |  | 6.18 | 6.21 | 6.23 | 6.25 | 6.28 | 6.30 | 7.3 | 7.5 | 7.7 | 7.9 | 7.12 | 7.14 | 7.17 | 94.90% |
| 11 | WACX | 4.19 | 4.21 | 4.23 | 4.26 | 4.28 | 4.30 | 5.3 | 5.5 | 5.8 | 5.10 | 5.12 | 5.14 | 5.17 | 5.19 |  | 5.21 | 5.24 | 5.26 | 5.28 | 5.31 | × | 6.5 | 6.7 | 6.9 | 6.11 | 6.14 | × |  | 6.18 | 6.21 | 6.23 | × | 6.28 | 6.30 | 7.3 | 7.5 | 7.7 | 7.9 | 7.12 | 7.14 | 7.17 | 92.30% |
| 12 | ZHLH | 4.19 | 4.21 | 4.23 | 4.26 | 4.28 | 4.30 | 5.3 | 5.5 | 5.8 | 5.10 | 5.12 | 5.14 | 5.17 | 5.19 |  | × | 5.24 | 5.26 | 5.28 | 5.31 | 6.2 | 6.5 | 6.7 | 6.9 | 6.11 | 6.14 | 6.16 |  | 6.18 | 6.21 | 6.23 | 6.25 | 6.28 | 6.30 | 7.3 | 7.5 | 7.7 | 7.9 | 7.12 | 7.14 | 7.17 | 97.40% |
| 13 | LIYY | 4.19 | 4.21 | 4.23 | 4.26 | 4.28 | 4.30 | 5.3 | 5.5 | 5.8 | 5.10 | 5.12 | 5.14 | 5.17 | 5.19 |  | 5.21 | 5.24 | 5.26 | 5.28 | 5.31 | × | 6.5 | 6.7 | 6.9 | 6.11 | 6.14 | 6.16 |  | 6.18 | 6.21 | 6.23 | 6.25 | 6.28 | 6.30 | 7.3 | 7.5 | 7.7 | 7.9 | 7.12 | 7.14 | 7.17 | 94.90% |
| 14 | FAHY | 5.3 | × | 5.8 | 5.10 | 5.12 | 5.14 | × | 5.19 | 5.21 | 5.24 | × | × | 5.31 | 6.2 |  | × | 6.7 | 6.9 | × | × | 6.16 | 6.18 | × | × | 6.25 | × | 6.30 |  | 7.3 | × | 7.7 | 7.9 | 7.12 | 7.14 | × | 7.19 | × | 7.24 | 7.26 | 7.28 | 7.30 | 66.70% |
| 16 | YUZQ | 5.3 | 5.5 | 5.8 | 5.10 | 5.12 | 5.14 | 5.17 | 5.19 | 5.21 | 5.24 | 5.26 | 5.28 | 5.31 | 6.2 |  | 6.5 | 6.7 | 6.9 | 6.11 | 6.14 | 6.16 | 6.18 | 6.21 | 6.23 | 6.25 | 6.28 | 6.30 |  | 7.3 | × | 7.7 | 7.9 | 7.12 | 7.14 | 7.17 | 7.19 | 7.21 | 7.24 | 7.26 | 7.28 | 7.30 | 97.40% |
| 19 | SURN | 5.3 | 5.5 | 5.8 | 5.10 | 5.12 | 5.14 | 5.17 | 5.19 | 5.21 | 5.24 | 5.26 | × | 5.31 | 6.2 |  | × | 6.7 | 6.9 | 6.11 | 6.14 | × | 6.18 | × | 6.23 | × | × | × |  | 7.3 | × | × | 7.9 | × | 7.14 | × | 7.19 | × | 7.24 | × | 7.28 | 7.30 | 66.70% |
| 24 | HOLL | 8.9 | 8.11 | 8.13 | 8.18 | 8.20 | 8.23 | 8.25 | 8.27 | 8.30 | × | 9.3 | 9.6 | 9.8 | 9.11 |  | × | × | 9.17 | 9.20 | 9.22 | 9.24 | 9.27 | 9.29 | × | 10.6 | 10.8 | 10.11 |  | 10.13 | × | 10.18 | 10.20 | 10.22 | 10.25 | 10.29 | 11.1 | 11.3 | 11.5 | 11.8 | 11.10 | 11.12 | 87.20% |
| 25 | XUSJ | 8.9 | × | 8.13 | 8.18 | 8.20 | 8.23 | 8.25 | 8.27 | 8.30 | 9.1 | 9.3 | 9.6 | × | 9.11 |  | × | 9.15 | 9.17 | 9.20 | 9.22 | 9.24 | 9.27 | 9.29 | × | 10.6 | 10.8 | 10.11 |  | 10.13 | 10.15 | × | 10.20 | 10.22 | 10.25 | 10.29 | 11.1 | 11.3 | 11.5 | 11.8 | 11.10 | × | 84.60% |
| 26 | SHYL | 8.9 | 8.11 | × | × | × | 8.23 | 8.25 | 8.27 | 8.30 | 9.1 | 9.3 | 9.6 | 9.8 | × |  | 9.13 | 9.15 | 9.17 | × | 9.22 | × | 9.27 | 9.29 | 10.4 | × | × | 10.11 |  | × | 10.15 | 10.18 | 10.20 | 10.22 | 10.25 | 10.29 | 11.1 | 11.3 | 11.5 | 11.8 | 11.10 | 11.12 | 76.90% |
| 28 | WAQZ | 8.9 | 8.11 | 8.13 | 8.18 | 8.20 | 8.23 | 8.25 | 8.27 | 8.30 | 9.1 | 9.3 | 9.6 | 9.8 | 9.11 |  | 9.13 | 9.15 | 9.17 | 9.20 | 9.22 | 9.24 | × | 9.29 | 10.4 | 10.6 | 10.8 | 10.11 |  | 10.13 | 10.15 | 10.18 | 10.20 | 10.22 | 10.25 | 10.29 | 11.1 | 11.3 | 11.5 | 11.8 | 11.10 | 11.12 | 97.40% |
| 29 | SUJJ | 8.9 | 8.11 | 8.13 | × | 8.20 | × | × | × | × | × | × | × | × | × |  | × | × | × | × | × | × | × | × | × | × | × | × |  | × | × | × | × | × | × | × | × | × | × | × | × | × | 10.30% |
| 30 | NIHF | 8.9 | × | 8.13 | 8.18 | × | 8.23 | 8.25 | × | 8.30 | 9.1 | × | × | 9.8 | 9.11 |  | × | 9.15 | 9.17 | × | 9.22 | 9.24 | 9.27 | × | 10.4 | 10.6 | × | 10.11 |  | 10.13 | × | 10.18 | 10.20 | 10.22 | 10.25 | × | 11.1 | 11.3 | 11.5 | 11.8 | × | × | 66.70% |
| 31 | JIFH | 8.9 | 8.11 | 8.13 | 8.18 | 8.20 | 8.23 | 8.25 | 8.27 | 8.30 | 9.1 | 9.3 | 9.6 | 9.8 | × |  | 9.13 | 9.15 | 9.17 | × | 9.22 | × | 9.27 | 9.29 | 10.4 | 10.6 | 10.8 | × |  | 10.13 | 10.15 | × | 10.20 | 10.22 | 10.25 | 10.29 | 11.1 | 11.3 | 11.5 | 11.8 | × | 11.12 | 84.60% |
| 34 | TALL | 9.27 | 9.29 | 10.4 | 10.6 | 10.8 | 10.11 | 10.13 | 10.15 | 10.18 | 10.20 | 10.22 | 10.25 | 10.29 | 11.1 |  | 11.3 | × | 11.8 | 11.10 | 11.12 | 11.15 | 11.17 | 11.19 | 11.22 | 11.24 | 11.26 | 11.29 |  | 12.1 | 12.3 | 12.6 | 12.8 | 12.10 | 12.13 | 12.15 | 12.17 | 12.20 | 12.22 | 12.24 | 12.27 | 12.29 | 97.40% |
| 40 | LISF | 9.27 | 9.29 | 10.4 | 10.6 | 10.8 | 10.11 | 10.13 | 10.15 | 10.18 | 10.20 | 10.22 | 10.25 | 10.29 | 11.1 |  | 11.3 | 11.5 | 11.8 | 11.10 | 11.12 | 11.15 | 11.17 | 11.19 | 11.22 | 11.24 | 11.26 | 11.29 |  | 12.1 | 12.3 | 12.6 | 12.8 | 12.10 | 12.13 | × | 12.17 | 12.20 | 12.22 | 12.24 | 12.27 | 12.29 | 97.40% |
| 42 | ZHLJ | 10.20 | 10.22 | 10.25 | 10.27 | 10.29 | 11.1 | 11.3 | 11.5 | 11.8 | 11.10 | 11.12 | 11.15 | 11.17 | 11.19 |  | 11.22 | 11.24 | 11.26 | 11.29 | 12.1 | 12.3 | 12.6 | 12.8 | 12.10 | 12.13 | 12.15 | 12.17 |  | 12.20 | 12.22 | 12.24 | × | 12.29 | 12.31 | 2023.1.2 | 2023.1.3 | 2023.1.5 | 2023.1.7 | 2023.1.10 | 2023.1.12 | 2023.1.14 | 97.40% |
| 44 | LISJ | 10.20 | 10.22 | 10.25 | 10.27 | 10.29 | 11.1 | 11.3 | 11.5 | 11.8 | 11.10 | 11.12 | 11.15 | 11.17 | 11.19 |  | 11.22 | 11.24 | 11.26 | 11.29 | 12.1 | 12.3 | 12.6 | 12.8 | × | × | 12.15 | 12.17 |  | 12.20 | × | 12.24 | 12.27 | 12.29 | 12.31 | 1.2 | 1.3 | 1.5 | 1.7 | 1.10 | 1.12 | 1.14 | 92.30% |
| 46 | YACF | 10.20 | 10.22 | × | 10.27 | 10.29 | 11.1 | 11.3 | 11.5 | 11.8 | 11.10 | 11.12 | 11.15 | 11.17 | 11.19 |  | 11.22 | × | 11.26 | 11.29 | 12.1 | × | 12.6 | 12.8 | 12.10 | × | 12.15 | 12.17 |  | 12.20 | 12.22 | 12.24 | × | × | 12.31 | 1.2 | 1.3 | × | 1.7 | × | 1.12 | 1.14 | 79.50% |
| 48 | LIGS | 10.20 | 10.22 | 10.25 | 10.27 | 10.29 | 11.1 | 11.3 | 11.5 | 11.8 | 11.10 | 11.12 | 11.15 | 11.17 | 11.19 |  | 11.22 | 11.24 | 11.26 | 11.29 | 12.1 | 12.3 | 12.6 | 12.8 | 12.10 | 12.13 | 12.15 | 12.17 |  | 12.20 | 12.22 | 12.24 | 12.27 | 12.29 | 12.31 | 1.2 | 1.3 | 1.5 | × | 1.10 | 1.12 | 1.14 | 97.40% |
| 49 | GAZH | 10.20 | 10.22 | 10.25 | 10.27 | 10.29 | × | 11.3 | 11.5 | 11.8 | 11.10 | 11.12 | 11.15 | 11.17 | 11.19 |  | 11.22 | 11.24 | 11.26 | 11.29 | 12.1 | 12.3 | 12.6 | 12.8 | 12.10 | 12.13 | 12.15 | × |  | × | × | 12.24 | 12.27 | 12.29 | 12.31 | 1.2 | 1.3 | 1.5 | 1.7 | 1.10 | 1.12 | 1.14 | 89.70% |
| 51 | LIYL | 10.20 | 10.22 | 10.25 | 10.27 | 10.29 | 11.1 | 11.3 | 11.5 | 11.8 | 11.10 | 11.12 | 11.15 | 11.17 | 11.19 |  | 11.22 | 11.24 | 11.26 | × | 12.1 | 12.3 | 12.6 | 12.8 | 12.10 | 12.13 | 12.15 | 12.17 |  | 12.20 | 12.22 | 12.24 | 12.27 | 12.29 | 12.31 | 1.2 | 1.3 | 1.5 | 1.7 | 1.10 | 1.12 | 1.14 | 97.40% |
| 54 | LISX | 10.20 | 10.22 | 10.25 | 10.27 | 10.29 | 11.1 | 11.3 | 11.5 | 11.8 | 11.10 | 11.12 | 11.15 | 11.17 | 11.19 |  | 11.22 | 11.24 | 11.26 | 11.29 | 12.1 | 12.3 | 12.6 | 12.8 | 12.10 | 12.13 | 12.15 | 12.17 |  | 12.20 | 12.22 | 12.24 | 12.27 | × | 12.31 | 1.2 | 1.3 | 1.5 | 1.7 | 1.10 | 1.12 | 1.14 | 97.40% |
| 56 | FEYJ | 10.20 | 10.22 | 10.25 | 10.27 | 10.29 | 11.1 | 11.3 | 11.5 | 11.8 | 11.10 | 11.12 | 11.15 | 11.17 | 11.19 |  | 11.22 | 11.24 | 11.26 | 11.29 | 12.1 | 12.3 | 12.6 | 12.8 | 12.10 | 12.13 | 12.15 | 12.17 |  | 12.20 | × | 12.24 | 12.27 | 12.29 | × | 1.2 | 1.3 | × | 1.7 | 1.10 | 1.12 | 1.14 | 92.30% |
| 57 | ZHLY | 10.20 | 10.22 | 10.25 | 10.27 | 10.29 | 11.1 | 11.3 | 11.5 | × | 11.10 | 11.12 | 11.15 | 11.17 | 11.19 |  | 11.22 | 11.24 | 11.26 | 11.29 | 12.1 | 12.3 | 12.6 | 12.8 | 12.10 | 12.13 | × | × |  | 12.20 | 12.22 | 12.24 | 12.27 | 12.29 | 12.31 | 1.2 | × | 1.5 | 1.7 | 1.10 | 1.12 | 1.14 | 89.70% |
| 61 | BACX | 10.20 | 10.22 | 10.25 | 10.27 | 10.29 | 11.1 | 11.3 | 11.5 | × | 11.10 | 11.12 | 11.15 | 11.17 | × |  | 11.22 | 11.24 | 11.26 | × | 12.1 | 12.3 | × | 12.8 | 12.10 | × | × | × |  | 12.20 | 12.22 | 12.24 | × | 12.29 | 12.31 | × | 1.3 | 1.5 | 1.7 | 1.10 | 1.12 | 1.14 | 76.90% |
| 62 | WAXX | 10.20 | 10.22 | 10.25 | × | × | × | 11.3 | × | × | × | × | 11.15 | × | × |  | × | × | × | × | × | × | × | × | × | × | × | × |  | × | × | × | × | × | × | × | × | × | × | × | × | × | 12.80% |
| 63 | MADH | 10.20 | 10.22 | 10.25 | 10.27 | 10.29 | 11.1 | × | × | 11.8 | × | 11.12 | 11.15 | × | 11.19 |  | 11.22 | × | 11.26 | 11.29 | 12.1 | × | × | 12.8 | 12.10 | × | 12.15 | 12.17 |  | × | 12.22 | 12.24 | × | 12.29 | 12.31 | × | 1.3 | 1.5 | × | 1.10 | × | 1.14 | 66.70% |
| 64 | YAXI | 10.20 | 10.22 | 10.25 | 10.27 | 10.29 | 11.1 | 11.3 | 11.5 | 11.8 | 11.10 | 11.12 | 11.15 | 11.17 | 11.19 |  | × | 11.24 | 11.26 | 11.29 | 12.1 | 12.3 | 12.6 | 12.8 | 12.10 | 12.13 | 12.15 | 12.17 |  | 12.20 | 12.22 | 12.24 | × | 12.29 | 12.31 | 1.2 | 1.3 | 1.5 | 1.7 | 1.10 | 1.12 | 1.14 | 94.50% |
